# Temperature Measurement of a Bullet in Flight

**DOI:** 10.3390/s20247016

**Published:** 2020-12-08

**Authors:** Corentin Kerampran, Tomasz Gajewski, Piotr W. Sielicki

**Affiliations:** 1 Higher National Institute of Professorship and Education, University of Poitiers, 15 Rue de l’Hôtel Dieu, TSA 71117, 86000 Poitiers, France; corentin.kerampran@etu.univ-poitiers.fr; 2 Institute of Structural Analysis, Poznan University of Technology, Maria Sklodowska-Curie Street 5, 60-965 Poznan, Poland

**Keywords:** temperature measurement, flying bullet, temperature analysis, bullet trajectory

## Abstract

This study answers a primary question concerning how the temperature changes during the flight of a bullet. To answer the question, the authors performed unique research to measure the initial temperatures of bullet surfaces and applied it to four kinds of projectiles in a series of field experiments. The technique determines the temperature changes on metallic objects in flight that reach a velocity of 300 to 900 m/s. Until now, the tests of temperature change available in the literature include virtual points that are adopted to ideal laboratory conditions using classic thermomechanical equations. The authors conducted the first study of its kind, in which is considered four projectiles in field conditions in which a metallic bullet leaves a rifle barrel after a powder deflagration. During this process, heat is partly transferred to the bullet from the initial explosion of the powder and barrel-bullet friction. In this case, the temperature determination of a bullet is complex because it concerns different points on the external surface. Thus, for the first time the authors measured the temperatures at different position on the bullet surface. Moreover, the authors showed that basic thermodynamic equations allow for the credible prediction of such behavior if the initial conditions are identified correctly. This novel identification of the initial conditions of temperature and velocity of flying bullets was not presented anywhere else up to now.

## 1. Introduction

Knowledge of real temperature changes of bullets during flight may have useful applications for many challenges. One of the challenges is related to forensic, judicial or military investigations. The temperature of a bullet upon striking its target can be important information for such investigations. Infrared imaging for “visualizing differences in temperature and/or emissivity of objects” is a used in forensic sciences expanding its possibilities [1]. For instance, scientific studies show that a heat generation and temperature increase can damage or reorganize the structure of DNA [2,3,4]. Therefore, crucial information for an investigation can be corrupted if the temperature of the bullet upon reaching the target is too high. Thus, it is useful to know the maximum temperature of a bullet during a shot. Knowledge of this maximum temperature would, for example, help to determine whether the DNA of an affected person has changed due to the temperature reached by the bullet. Furthermore, a systematic study of the temperature evolution for various bullets would help to determine the caliber and other characteristics of the projectile during various investigations.

In the literature, there are almost no studies devoted to temperature evolution of a bullet in flight, nor experimental techniques for how to measure it. If these studies are published, they are classified information. Such experiments remain difficult to carry out due to the small size and high speed of a bullet during a flight. A testing protocol would require the use of high-precision equipment, capable of capturing the passage of a bullet within these demanding conditions. Moreover, temperature acquisition at high-speed is a challenging task, as shown by Huiping et al. [5] and Fang et al. [6].

As this study is the first in the field, it is impossible to find papers that directly address the same subject matter. Few studies have conducted temperature measurements for high-speed aerial objects. Rather, the literature, theoretical or numerical, is related to the aerodynamic effects of aircraft or missiles, namely, heating, coolin or infrared characteristics.

In an analysis by Gunduz et al. [7], the objective of the study was to measure temperature evolution during a flight of a system located under the wing of an aircraft. Analytical predictions were obtained using equations from the scientific literature. Subsequently, experimental measurements at different altitudes and flight speeds were made, and the results obtained were close to the analytical predictions.

Many recent techniques of thermal measurement may be found in the literature. In the paper of Jaremkiwicz et al. [8], a new technique for measuring transient rapid changes in temperature was presented. As reported, this technique allows for a more accurate temperature determination than typical devices in conventional and nuclear power plants, which have high thermal inertia. Nonetheless, the technique is not applicable for flying objects. Moreover, Goumopoulos [9] proposed the thermal technique of delivering a high accuracy of the measurements. Moreover, a recording device is cheap and is already applied in agriculture and medicine. The sensors for similar purposes were presented in [10]. They provide a cheap temperature measurement for everyday life, agriculture or intelligent health devices. These techniques require specific laboratory conditions or ensuring measurements in a static position, thus they are not applicable at the testing field, where the bullets should be tested. In a related paper, Szklarski, Świderski, and Machowski [11] presented a concept for an experimental setup to test the heating of the missile body at flight. The measurements were carried out in a wind tunnel. Four missiles were selected in order to test whether some typical designs would have significantly different flight velocities and aerodynamics. In addition to the measurements of velocities and aerodynamics, a numerical study of the four missiles was carried out to determine the areas in which the heat increase was the most important.

If no wind tunnel could be used, for instance due to fluctuating pressure fields, the interesting concept of surface pressure measurements of free flight objects via paint were presented by Kurihara et al. [12] This technique may be used to different free flight objects; it is not limited to a specific flow regime or model type.

In a paper by Jianwei and Qiang [13], the skin temperature of an aircraft during flight was simulated for developing stealth technology. The simulations were performed according to computational fluid dynamics. The authors reported that aerodynamic heating plays a crucial role, but no experimental data were presented in the study. In addition, in the literature one can find papers regarding theoretical and numerical analyses on aerodynamic heating and radiation from the environment for aircraft skin [13,14].

In a paper by Abukhshim, Mativenga, and Sheikh [15], the objective was to determine the maximum temperature and the temperature distribution along the rake face of a cutting tool. They demonstrated an analytical prediction to estimate the heat generation in metal cutting processes. Experimental measurements were conducted using a camera FLIR ThermaCAM SC3000^©^ to determine the temperature during machining sessions at various cutting speeds. A related analysis by Schreivogel et al. [16] attempted to conduct measurements through the use of high-speed cameras and a pulsed, high-speed UV laser to excite particles. In addition, thermographic particle image velocimetry was used to investigate the flow emanating from cooling holes in a closed-loop, optically accessible wind tunnel facility.

In Celik et al. [17], the objective of the study was to investigate heat transfer between a steel strip and a rotating heat pipe. To determine the heat transfer between two moving surfaces, the problem was decoupled into two parts: gas entrainment and heat transfer. Experimental measurements were executed on a rotating heat pipe by varying the strip thickness, specific tension and strip velocity.

Innovative thermal measurement techniques could not only be used in ballistics, but in related dynamic areas too. Those techniques could be adopted in scientific studies regarding blast studies [18,19,20] but also in research regarding constitutive frameworks, such as [21,22]. Finally, the ballistic studies could be enhanced by thermal techniques, for instance in [23]. Furthermore, the industrial processes may be controlled or inspected by thermal innovative techniques, like in [24,25], in which the infrared techniques were used to continuously measure the molten iron temperature and provide robust data to control a blast furnace.

**Figure 1:**
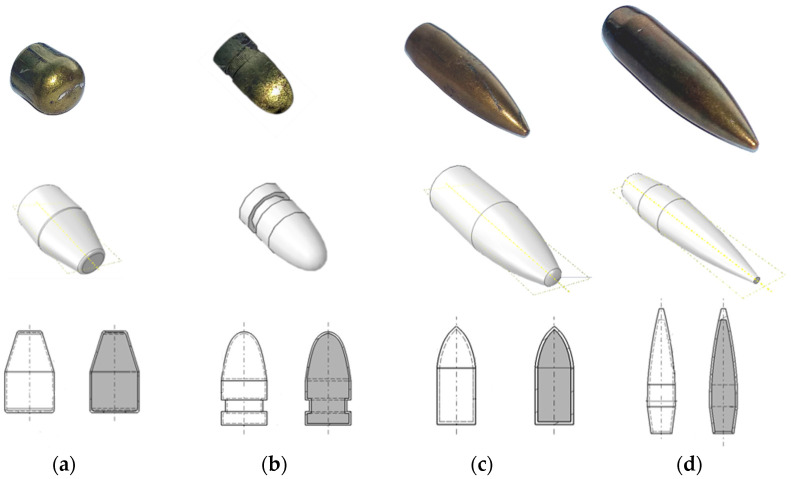
The real and virtual geometry of bullets used in the experimental study: (**a**) 9 mm × 19 mm Parabellum, (**b**) 9 mm × 29 mm (0.38 “Special”), (**c**) 7.62 mm × 39 mm (AK ammunition) and (**d**) 7.62 mm × 51 mm (0.308 Winchester ammunition).

Given the lack of literature that specifically addresses the topic of the present paper, we reveal the results obtained experimentally and compare them with analytical predictions. To obtain generalizable results and deeper insights, the tests were carried out on four different caliber. As described in Figure 1, the following calibers were tested: the 9 mm × 19 mm Parabellum, 9 mm × 29 mm (revolver bullet, i.e., 0.38 “Special”), 7.62 mm × 39 mm (AK ammunition) and 7.62 mm × 51 mm (0.308 Winchester ammunition). All calibers had different geometries and masses. Bullet masses were equal to 8 g, 9 g, 7.5 g, and 7.5 g, respectively, and these values were used in further calculations. Technical data were obtained from the manufacturers of the bullets. All bullets used in the study were composed of two parts, a jacket of copper/zinc alloy and a lead body.

The overall objective of our study was to experimentally measure the temperature of bullets in flight; to do so, we conducted tests by means of a thermal camera and a high velocity camera. Our paper has two objectives: the primary goal is to measure bullet temperatures in flight for different calibers. A secondary objective was to ascertain the temperature rise for various parts of different bullets during flight. Moreover, to acquire a deeper understanding of the aerodynamic heating of the bullets in flight, an analytical prediction was proposed with a dedicated testing protocol. The unique results obtained provide an insight into the temperature change of bullets during flight.

## 2. Materials and Methods

### 2.1. Analytical Background

Heat transfer in ballistics has been explored for many years. The governing equations are known from classical thermomechanics and are taught in well-known textbooks. For example, Nellis and Klein [26] state that the analytical relationship presented in Equation (1) allows for determination of the drag force caused by high velocity on moving objects in the air. In the case considered, the bullet velocity u is balanced by the drag force F:

(1) $$M\;\frac{du}{dt}=-F,$$

where M represents the mass of the bullet. Here, the actual values were used, but for sphere M is calculated by the following equation:

(2) $$M=\frac{4\pi }{3}{\left(\frac{D}{2}\right)}^{3}\;\rho$$

where D is the diameter of the bullet and ρ is the density of the bullet material. After manipulation of Equation (1) one may obtain the time rate of change of the bullet velocity u:

(3) $$\;\frac{du}{dt}=-\frac{F}{M}.$$

The drag force of the bullet F is computed by:

(4) $$F=C_{D}\frac{\rho_{air}u^{2}}{2}\frac{\pi D^{2}}{4},$$

where $C_{D}$ is the drag coefficient, $\rho_{air}$ is the density of air and D is the diameter of a flying spherical object. Drag coefficients in the study were computed according to Gavre drag functions, namely G1 and G7 models. G1 was used for 9 mm × 19 mm Parabellum and 9 mm × 29 mm (0.38 “Special”), while G7 was used for 7.62 mm × 39 mm (AK) and 7.62 mm × 51 mm (0.308 Winchester). As in Nellis and Klein [26], the bullet can be modelled as a sphere; thus, D is taken as the bullet diameter.

The time rate of change of the distance travelled by the bullet x is equal to the velocity, as seen in the following equation:

(5) $$\;\frac{dx}{dt}=u.$$

Furthermore, as shown in Nellis and Klein [26], the temperature of the bullet is governed by an energy balance and is characterized by the following equation:

(6) $$Mc\frac{dT}{dt}=\bar{h}\;\pi D^{2}\left(T_{\infty }-T\right),$$

where c represents the specific heat capacity of the bullet, T is its temperature, and $\bar{h}$ is the average heat transfer coefficient. Equation (6) may be rearranged to provide the instantaneous temperature rate of change:

(7) $$\frac{dT}{dt}=\frac{\bar{h}\pi D^{2}}{Mc}\left(T_{\infty }-T\right).\;$$

The average heat transfer coefficient $\bar{h}$ takes the following form:

(8) $$\bar{h}=\frac{Nu_{D}k_{air}}{D},$$

where, $Nu_{D}$ is the Nusselt number and $k_{air}$ is the heat conductivity of air [W/(m K)]. The heat conductivity [27] is introduced as:

(9) $$k_{air}=0.02626{\left(\frac{T}{300}\right)}^{0.8646}.$$

The Nusselt number $Nu_{D}$ (after Nellis and Klein [26]) is introduced by:

(10) $$Nu_{D}=2+\left(0.4Re_{D}^{\frac{1}{2}}+0.06Re_{D}^{\frac{2}{3}}\right)Pr_{}^{\frac{2}{5}},$$

where $Re_{D}$ is Reynolds number of air and Pr is the Prandtl number of air. The Reynolds number of air is obtained by:

(11) $$Re_{D}=\frac{\rho_{air}uD}{\mu_{air}},$$

where $\rho_{air}$ is air density, D is a characteristic linear dimension (bullet diameter) and $\mu_{air}$ is air viscosity. The Prandtl number of air is taken as:

(12) $$Pr=\frac{c_{p}\mu_{air}}{k},\;$$

where $c_{p}$ is the specific heat capacity.

Air density is postulated as:

(13) $$\rho_{air}=\frac{p}{RT},$$

where p is an air pressure, *R* = 287.058 J/(kgK) and is a specific gas (air) constant.

Air viscosity $\mu_{air}$, from Sutherland’s law [28], is expressed by:

(14) $$\mu_{air}=\mu_{air0}{\left(\frac{T}{T_{0}}\right)}^{\frac{3}{2}}\frac{T_{0}+S}{T+S},$$

where S=110.55 K and $\mu_{air}=1.76\times 10^{-5}$ kg/ms.

Finally, to determine the position, velocity and temperature of the bullet in the time domain, the following system of equations must be solved [26]:

(15) $$\{\begin{array}{c} \frac{dx}{dt}=u,\\ \frac{du}{dt}=\frac{C_{D}\rho_{air}u^{2}\pi D^{2}}{8M}\\ \frac{dT}{dt}=\frac{\bar{h}\pi D^{2}}{Mc}\left(T_{\infty }-T\right). \end{array},$$

**Figure 2:**
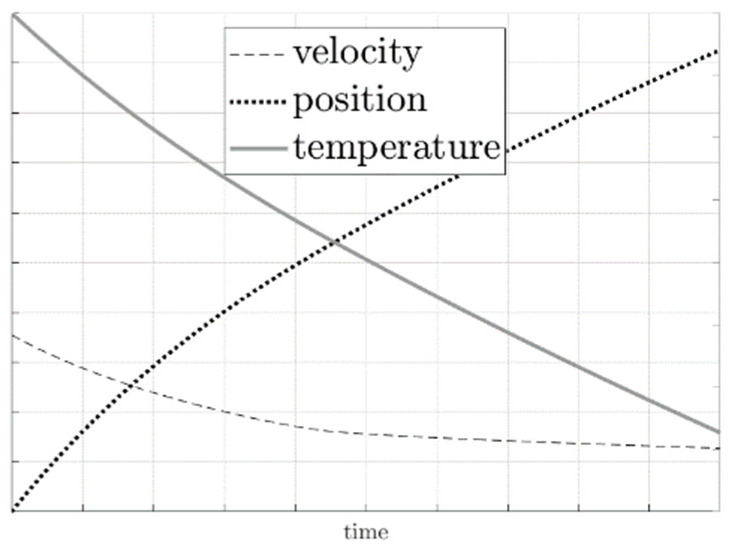
Graphical representation of a typical solution of a system of Equation (15).

In the present study, we employed a Runge-Kutta method of IV in order to solve the system of equations for the selected bullets considered in the study. Figure 2 presents a typical solution reproduced from the system of Equation (15).

### 2.2. Experimental Measurement

The aim of this experimental setup was to measure the temperature and velocity of the bullet during flight at various distances. To have a more globally applicable understanding of the variation of a bullet, temperature-in-flight tests were conducted for different calibers of bullets with differing geometries. Four bullet calibers were tested, namely 9 mm × 19 mm Parabellum, 9 mm × 29 mm (revolver bullet, i.e.,0.38 “Special”), 7.62 mm × 39 mm (AK ammunition) and 7.62 mm × 51 mm (0.308 Winchester ammunition), as described in Figure 1.

**Figure 3:**
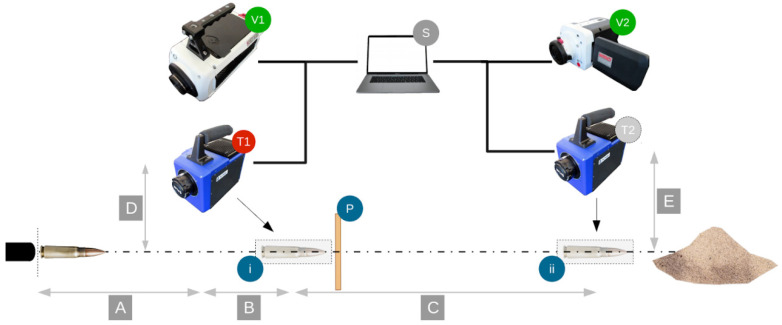
The scheme of the setup of field-testing devices. (**V1**,**V2**)—velocity high-speed camera, (**T1**,**T2**)—thermal high-speed camera, (**i**/**ii**)—registration frames, (**P**)—target panel, (**S**)—computer with software.

To measure the magnitudes of bullet temperature and velocity, a new testing protocol was designed. The scheme of the setup for testing devices is presented in Figure 3. Measurements were done at frame (i) and frame (ii). Frame (i) is the distance of A + B from the shooting position to the beginning. A represents the distance between the shooter and the position of the first cameras. B represents the distance between the thermal camera, T1, and the frame (i). The second measurement was done at frame (ii), namely A + B + C distance from the shooter before reaching the sand state. C represents the distance between the two frames (i) and (ii).

The bullets were recorded in two positions to compare results at two different times. Such an approach allowed us to determine the temperature evolution for each shooting distance. Two high-speed cameras and a high-speed thermal camera were used to perform the tests. A Phantom V711 © high-speed camera, labelled as V1, was used to measure bullet velocity in the first part of the shot at A + B distance. Phantom V711© allows to record the movie with 7530 frames per second (FPS) at its maximal resolution, i.e., 1280 × 800 and 680,000 FPS at a resolution of 128 × 32. The second high-speed camera used was a MIRO 320S—V2©, which was positioned near the sand state to measure the velocity of the bullet at the end of the shot. MIRO 320S © allows recording of the movie with 1380 FPS at the resolution of 1920 × 1200, with resolution reduced to 64 × 8, the FPS equals 325,000. A high-speed thermal camera FLIR SC7000© T1/T2 with ALTAIR© software (S) was used to measure the temperature of the bullet in flight. FLIR SC7000© allows recording with 35,000 FPS for resolution of 640 × 512, the maximal thermal accuracy equals 17 mK. The thermal camera position was crucial to observe the bullet during the shot due to the small dimensions of the camera frame and limited time resolution. The window resolution was equal to 320 × 20/208 × 80 px, while the time resolution was equal to 3200 and 230,000 fps for thermal and velocity cameras, respectively. The exposure time in the thermal camera was to set the lowest possible value—10 µs, which was used in all of the results presented. Due to such values of the exposure time and a low resolution, we greatly increased the number of shots. This enabled the possibility to neglect all of those records which gave unsatisfactory results with blurring or partial volume effects. The length of the observed bullet trajectory was about 10–15 cm for both cameras. The measurements at positions T1 and T2 were made by the same device; the experiments were performed several times with the thermal camera at T1 or T2. The principle of velocity measurement was adopted from our recent work [29,30], in which the reader would find more details about it.

In Figure 3, the distances D and E represent the location on the side, perpendicular to different cameras in relation to the trajectory of the shot. D was equal to 1.5 m, while E was equal to 2 m. All shots were recorded with Altair computer software—a tool dedicated to analyzing recorded data. Four series of shots were conducted for each caliber selected for the study. In each series, 10 to 25 shots were fired, depending on the caliber. Most of the shots were properly recorded (depending on the particular type of bullets about 60–80%). Due to the limited frame size and time resolution of the devices, a minor number of shots were missing and unable to be recorded.

To obtain results with a higher accuracy, several special techniques were adopted. Those techniques included a proper setup (distances and framing), bullet preparation and a testing device calibration. To ensure the bullet trajectory went through the camera frames (i) and (ii), a shooting target was included. An active shooter was employed to perform the shooting. A panel with a shooting sheet of paper served as the target (P). The perforation of the paper causes negligible friction and thus had no impact on the temperature of the bullet surface or the bullet itself. Moreover, the sheet of paper did not influence the trajectory of the bullet.

**Figure 4:**
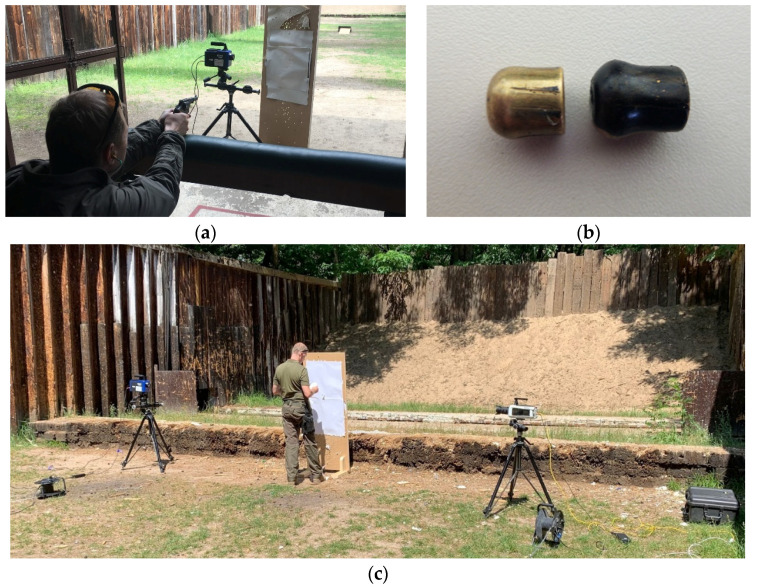
(**a**) Shooter aiming during field tests, (**b**) revolver bullets as received (shiny) and painted black, (**c**) viewing field landscape with testing camera.

The thermal camera was positioned so that its viewing window was positioned on the trajectory of the bullet. The shooter was 3 m away from the target (A = 2 m, B = 1 m, C = 40 m), as seen in Figure 4a. It should be underlined that the distance A should be a large enough field of observation to avoid the intrusion of smoke and dust effects that arise from releasing a bullet from a barrel (depending on the gun or bullet, the distance A may differ). Furthermore, since the thermal camera is sensitive to reflections and shiny surfaces, the metallic reflections of the bullets could compromise the results obtained. Thus, to avoid the shining effect, the bullets were covered with a black marker to give them a dark matte colour, as shown in Figure 4b.

**Figure 5:**
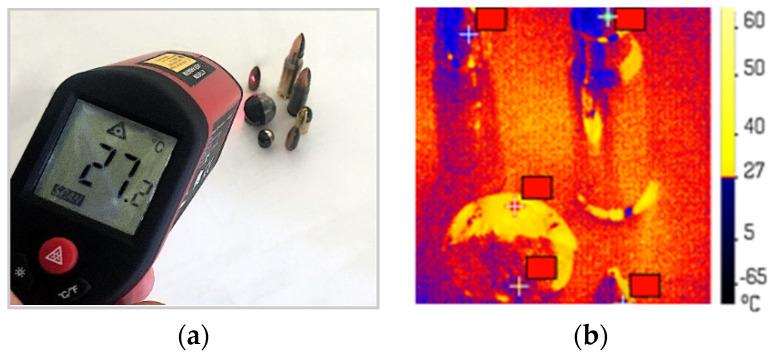
Emissivity determination: (**a**) infrared thermometer measurement, and (**b**) thermal camera recording for the correct emission ratio.

To obtain more accurate results, our surface temperature determination technique required the correct value of the surface emissivity. In this study, a calibration was carried out by using an infrared thermometer before the recording of the bullet temperature in flight was made (see Figure 5a). First, the temperatures of the individual bullets were measured using an infrared thermometer. Second, the same values were obtained in the thermal camera by changing the value of the emissivity, as shown in Figure 5b. The correct magnitude was obtained by a trial and error inverse method. Emissivity was determined to be 0.7. At that time, the initial temperature of the bullets were measured by infrared thermometer and by thermal camera and the values measured were the same. Finally, the default value of the emissivity from the Altair computer software was replaced by the value obtained in the calibration procedure (0.7). Similar values were used in the paper of Chybinski et al. [31] and may be found in the classical handbooks on infrared imaging [32,33,34,35].

## 3. Results and Discussion

**Figure 6:**
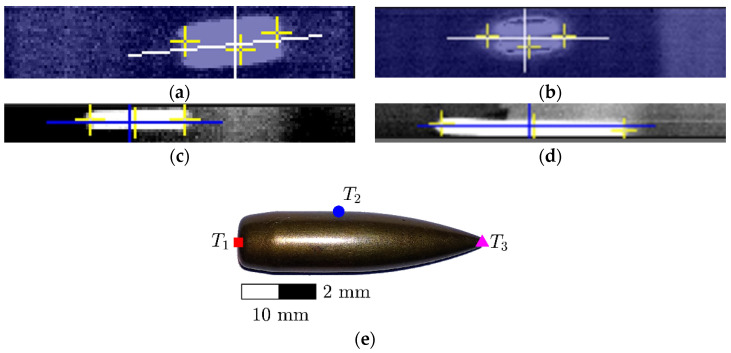
The examples of captured frames of (**a**) 9 × 19 mm Parabellum, (**b**) 9 × 29 mm, (**c**) 7.62 × 39 mm, and (**d**) 7.62 × 51 mm during temperature measurement by thermal camera (right after leaving the barrel at the distance of A + B). (**e**) Measurement point locations are labelled by T_i_, i = {1, 2, 3}.

Selected data recorded during all tests are presented in tabular form in Appendix A. Table A1, Table A2, Table A3 and Table A4 in Appendix A include velocity V_I_ and temperatures T_I,1_, T_I,2_, and T_I,3_ recorded at frame (i). Temperatures T_I,1_, T_I,2_, and T_I,3_ were measured at the bullet nose, side and rear, respectively. Part of the results had to be classified as secret due to the requirements of the government agency which financed the research presented. Thus, the measurements of V_II_ and T_II,1_, T_II,2_, and T_II,3_ at frame (ii) were not included. For the four calibers, namely 9 × 19 mm, 9 × 29 mm, 7.62 × 39 mm, and 7.62 × 51 mm, 6, 11, 23, and 4 shots were properly recorded, respectively. Examples of frames captured during tests by the FLIR thermal camera for each caliber are presented in Figure 6a–d. Furthermore, for clarity, the location of the temperature measurement points (T_i_, I = {1, 2, 3}) are shown in Figure 6e.

**Figure 7:**
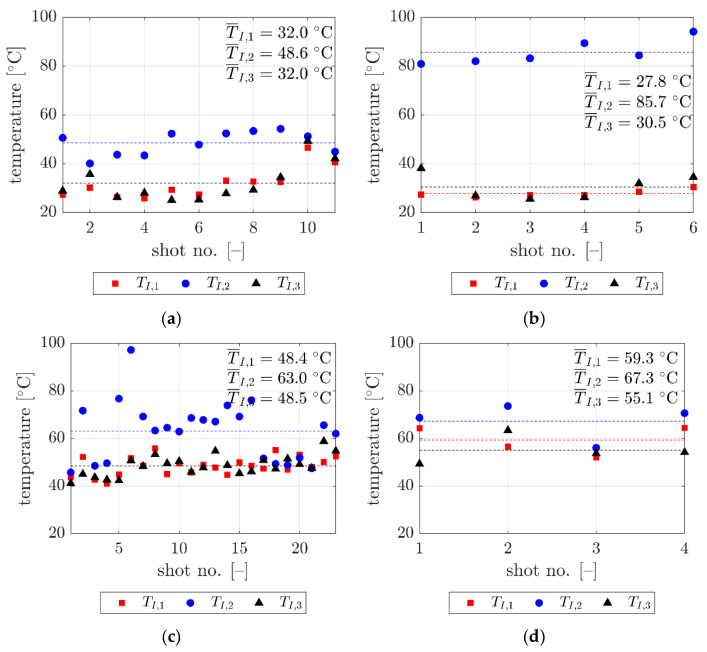
Temperatures measured at frame (i) for the tested types of bullets ((**a**) 9 mm × 19 mm Parabellum, (**b**) 9 mm × 29 mm 0.38 “Special”, (**c**) 7.62 mm × 39 mm AK, and (**d**) 7.62 mm × 51 mm 0.308 Winchester) and at different parts of the bullet (T_I,1_, T_I,2_, and T_I,3_). Mean values are marked by dashed lines.

**Figure 8:**
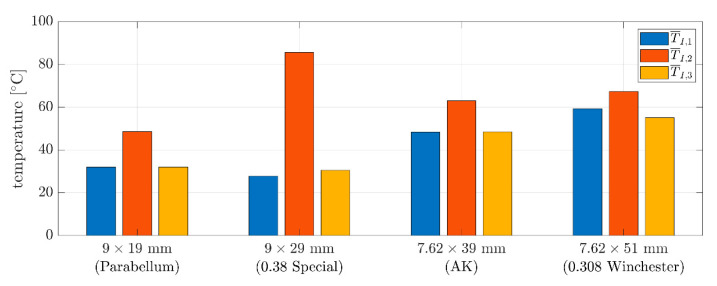
Average temperatures measured after leaving the barrel obtained for a particular type of bullet.

Temperature data from Appendix A, Table A1, Table A2, Table A3 and Table A4, are summarized in Figure 7. In the plots, the raw data obtained during the experiments are presented. These data are represented by the red squares for the rear temperatures (T_1_), blue circles show side temperatures (T_2_) and the black marks show the nose temperatures (T_3_) (see Figure 6e). The dispersion of the data is relatively low. The mean values were marked by dashed lines in the same color as the pointers (i.e., squares, circles, and crosses) and, for better clarity, are presented in Figure 8 as bar plots.

For the four different types of bullets, the highest temperatures were seen from the side of the bullet. The temperatures of the rear of the bullets were close to the temperatures of the nose. The largest gap between the side temperature and the temperature of the nose and the rear was for the 9 × 29 mm bullet, which measured a difference of about 55 °C. This phenomenon can be explained by the unique geometry of this bullet; for example, it is the only bullet that has a machined groove. In other cases, the same difference (side temperature versus rear and nose temperature) was about 11–17 °C. Furthermore, the shorter bullets (i.e., 9 × 19 mm, 9 × 29 mm) had nose and rear temperatures that were 15–25 °C lower than their longer counterparts (i.e., 7.62 × 39 mm, 7.62 × 51 mm). The side temperature differences for the different types of bullets were, therefore, inconsistent with each other.

**Figure 9:**
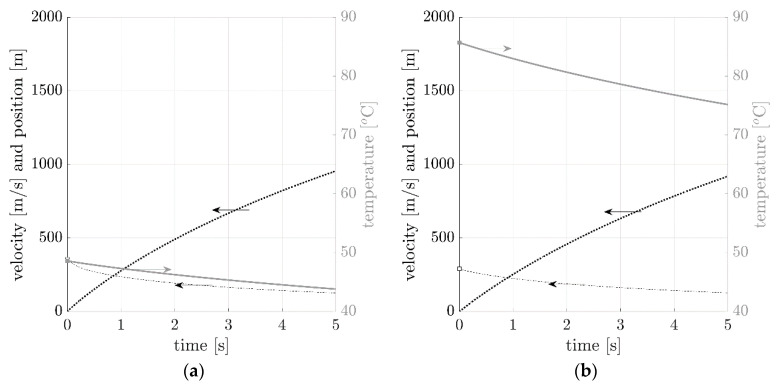

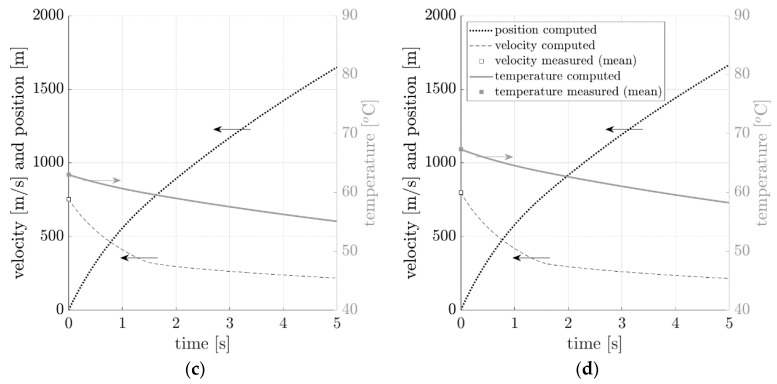
Temperature, velocity, and position plots for all bullets considered: (**a**) 9 mm × 19 mm Parabellum, (**b**) 9 mm × 29 mm (0.38 “Special”), (**c**) 7.62 mm × 39 mm (AK ammo), and (**d**) 7.62 mm × 51 mm (0.308 Winchester) obtained from a system of Equation (15).

In Figure 9, the relationship between velocity (dashed line), temperature (continuous line), and position (dotted line) are presented. The plots were computed for all bullets using the system of Equation (15), with the initial temperature and velocity taken from the experiments conducted in this study. Considering one of the example, i.e., AK ammo with 7.62 mm × 39 mm bullet, the average initial temperature of the bullet was 63 °C. The average initial velocity was equal to 753 m/s (see Appendix A, Table A3). The bullet leaving the barrel is hot and cools down over the course of the flight. This phenomenon is apparent on the graph given that there is a decrease in temperature during the flight. After 5 s, the temperature decrease predicted was approximately 8 °C. The distance travelled was predicted to be about 1650 m. It should be underlined that no vertical (ground) limit was assumed in the system of Equation (15). The bullet velocity also decreased to about 220 m/s after 5 s. Although 5 s appears to be a long period for such phenomena, it is included to show the non-linear character of the position, velocity, and temperature plots.

Similar effects may be observed in computations for all bullets. Apart from the obvious decrease in the velocity, the bullet temperature also decreased. After 5 s of undisturbed flight, the bullet temperature predicted decreased by 4.8 °C, 10.5 °C, 7.9 °C and 9.1 °C, respectively (see Figure 9). Velocity decreased, but the relative differences between the bullets were greater than for temperatures. For 9 mm × 19 mm Parabellum and 9 mm × 29 mm (0.38 “Special“) bullets the velocity drops were 230 m/s and 160 m/s, respectively; while for 7.62 mm × 39 mm (AK ammunition) and 7.62 mm × 51 mm (0.308 Winchester) bullets they were equal to 540 m/s and 580 m/s, respectively—more than 2.5 times bigger.

Since the study presented here considered a complex experimental technique to determine the initial condition for the thermomechanical equations, it has a few limitations. It is worth underlining here that in the system of thermomechanical equations, the bullets were modelled as the spheres. Moreover, the drag coefficients were adopted from G1 and G7 models used in ballistics, but were not determined experimentally or numerically for the particular shape of the bullet. Furthermore, the determination of the emission ratio due to its manual character has a moderate level of uncertainty.

## 4. Conclusions

Experimental tests are crucial for determining the proper boundary conditions of a problem. This paper presents the innovative temperature measurements of different bullets during flight. In our research, the bullets of 9 mm × 19 mm Parabellum, 9 mm × 29 mm (revolver bullet, i.e.,0.38 “Special”), 7.62 mm × 39 mm (AK ammunition), and 7.62 mm × 51 mm (0.308 Winchester ammunition) were considered. The study, as the first of its kind, demonstrated that due to differing masses of bullets, the thermal properties differ during flight. For the first time in the literature it was demonstrated that different temperatures were recorded at different places on the bullet’s surface. We also describe how plots of temperature, velocity, and position were obtained by mixing experimental and theoretical approaches. The temperatures observed for different bullets were from 25 °C up to 90 °C.

Moreover, we designed and described the technical details for a new measurement technique for determining temperature during flight. The technique requires a high-speed velocity and thermal camera and may be adopted to determine the thermal properties of other types of bullets. In comparing the results of different bullets, we add to the existing literature on the topic of bullet temperature and its evolution during flight, depending on geometry and mass. Moreover, the knowledge acquired through our study may be applied to other areas and disciplines such as forensic, judicial, or military investigations and analysis.

## Appendix A

**Table A1:** Velocities and temperatures registered for the 9 × 19 mm Parabellum bullet—frame (i).

Test Number	V_I_ (m/s)	T_I,1_ (°C)	T_I,2_ (°C)	T_I,3_ (°C)
1	360	27.4	50.6	28.9
2	354	30.2	40.1	35.7
3	358	26.3	43.7	26.3
4	354	25.8	43.4	28.0
5	356	29.3	52.3	25.1
6	359	27.4	47.8	25.3
7	353	33.1	52.4	27.8
8	354	32.7	53.4	29.3
9	353	32.5	54.3	34.4
10	350	46.6	51.2	49.3
11	352	40.6	44.9	42.1
Average	355	32.0	48.6	32.0

**Table A2:** Velocities and temperatures registered for the revolver bullet (9 × 29 mm, 0.38 “Special”)—frame (i).

Test Number	V_I_ (m/s)	T_I,1_ (°C)	T_I,2_ (°C)	T_I,3_ (°C)
1	295	27.4	80.9	38.1
2	293	26.2	82.0	26.9
3	293	27.1	83.2	25.6
4	288	27.1	89.4	26.2
5	288	28.5	84.4	31.9
6	285	30.4	94.1	34.5
Average	290	27.8	85.7	30.5

**Table A3:** Velocities and temperatures registered for the 7.62 × 39 mm (AK ammunition) bullet—frame(i).

Test Number	V_I_ (m/s)	T_I,1_ (°C)	T_I,2_ (°C)	T_I,3_ (°C)
1	756	43.6	45.7	41.1
2	745	52.3	71.7	45.0
3	750	42.6	48.5	43.6
4	749	41.0	49.6	42.6
5	756	44.8	76.7	42.3
6	748	51.6	97.2	50.7
7	756	48.6	69.2	48.3
8	748	55.8	63.3	53.3
9	757	45.0	64.5	49.5
10	754	49.5	62.9	50.4
11	753	45.7	68.6	45.9
12	754	48.9	67.8	47.7
13	754	47.7	67.1	54.7
14	754	44.7	73.9	48.7
15	753	49.9	69.2	45.3
16	754	48.5	76.1	46.1
17	754	47.3	51.6	50.8
18	752	55.1	49.3	47.2
19	758	46.9	48.8	51.5
20	753	53.1	51.9	49.1
21	756	48.0	47.4	47.5
22	753	50.1	65.6	58.8
23	754	52.4	62.0	54.7
Average	753	48.4	63.0	48.5

**Table A4:** Velocities and temperatures registered for the 7.62 × 51 mm (0.308 Winchester ammunition) bullet—frame(i).

Test Number	V_I_ (m/s)	T_I,1_ (°C)	T_I,2_ (°C)	T_I,3_ (°C)
1	798	64.3	68.7	49.3
2	796	56.5	73.6	63.4
3	802	52.0	56.1	53.7
4	799	64.4	70.6	54.2
Average	799	59.3	67.3	55.1
